# Liquid biopsy for cancer management: a revolutionary but still limited new tool for precision medicine

**DOI:** 10.1515/almed-2020-0009

**Published:** 2020-04-27

**Authors:** María Arechederra, Matías A. Ávila, Carmen Berasain

**Affiliations:** Hepatology Program, CIMA, University of Navarra, Pamplona, Spain; Instituto de Investigaciones Sanitarias de Navarra-IdiSNA, Pamplona, Spain; CIBERehd, Instituto de Salud Carlos III, Madrid, Spain; Hepatology Program, CIMA, University of Navarra, Avda. Pio XII, n55, 31008, Pamplona, Spain

**Keywords:** circulating biomarkers, circulating tumor cells (CTCs), circulating tumor DNA (ctDNA), personalized medicine, tumor circulome

## Abstract

The term liquid biopsy is used in contraposition to the traditional “solid” tissue biopsy. In the oncology field it has opened a new plethora of clinical opportunities as tumor-derived material is shedded into the different biofluids from where it can be isolated and analyzed. Common biofluids include blood, urine, saliva, cerebrospinal fluid (CSF), pleural effusion or bile. Starting from these biological specimens several analytes can be isolated, among which we will review the most widely used: circulating tumor cells (CTCs), circulating tumor DNA (ctDNA), circulating tumor RNA (ctRNA), proteins, metabolites, and exosomes. Regarding the nature of the biomarkers it will depend on the analyte, the type of tumor and the clinical application of the liquid biopsy and it includes, somatic point mutations, deletions, amplifications, gene-fusions, DNA-methylated marks, tumor-specific miRNAs, proteins or metabolites. Here we review the characteristics of the analytes and the methodologies used for their isolation. We also describe the applications of the liquid biopsy in the management of patients with cancer, from the early detection of cancers to treatment guidance in patients with advanced tumors. Finally, we also discuss some current limitations and still open questions.

## Introduction

Even before the definition of the term biomarker by a consensus panel at the WHO in Geneva in 2001 as “any substance, structure, or process that can be measured in the body or its products and influence or predict the incidence of outcome or disease” [1], the diagnosis and prognosis of multiple diseases had relied on the identification of different biomarkers in blood tests. Well-known examples are for instance the determination of the levels of different metabolites such as glucose or cholesterol; the presence of enzymes such as transaminases coming from dying hepatocytes; or the levels of monoclonal immunoglobulins, carcinoembryonic antigen (CEA), alfa-fetoprotein (AFP), prostate specific antigen (PSA), cancer antigen 125 (CA 125) or human chorionic gonadotropin (HCG) in the bloodstream [2], [3]. All these biomarkers have been commonly used with variable sensitivity and specificity rates to diagnose diabetes, liver diseases or the presence of myeloma, colon, liver, prostate, ovarian or germ-cell tumors.

However, in the oncology field these biomarkers in biological fluids have shown limited reliability [4]. In addition, during the last decades the large amount of high throughput molecular data obtained together with the progress of pharmacogenomics have demonstrated that it is required to characterize the molecular profile of the tumor for tailoring treatment regimens, the so-called precision medicine, monitoring therapy responses and detecting the emergence of therapy resistance [5]. Importantly, it is also known now that the genetic landscape of tumors is spatially heterogeneous and temporally dynamic [6], [7].

In this sense, the traditional method of tumor sampling by tissue biopsy has several limitations, including the fact that in some cases tumor accessibility is difficult and performing a biopsy may not be possible. In addition, given its invasive nature, tissue biopsies can pose risks to the patient what will also limit longitudinal monitoring. Importantly, tissue biopsies may present sample bias as they provide the tumor picture at a single location missing spatial tumor genetic heterogeneity and impacting in accuracy and sensitivity [6].

During the last years, the effort of multiple groups and the development of different sensitive technologies have allowed the advance of non-invasive methodologies to detect tumor-derived material in biofluids to be used for cancer diagnosis, prognosis, monitoring, and therapy guidance [8], [9], [10], [11], [12], [13].

**Figure 1:**
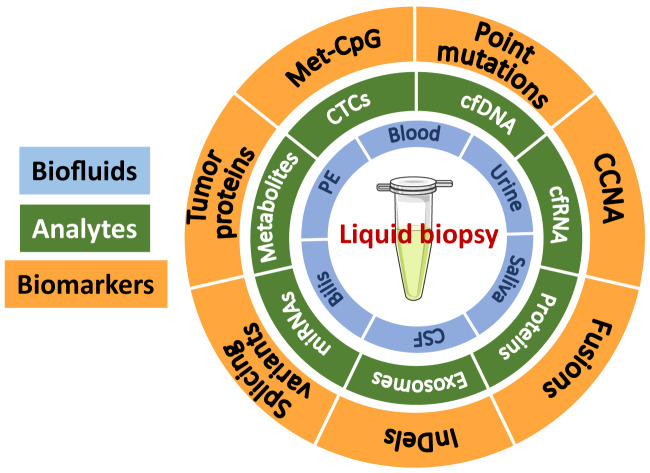
Biofluids, analytes and biomarkers encompassed in liquid biopsy. Met-CpG: methylated CpGs (dinucleotide C-G). CCNA: chromosomal copy number aberrations; InDel: insertions and deletions; CTCs: circulating tumor cells; cfDNA: cell-free DNA; cfRNA: cell-free RNA; miRNAs: microRNAs; PE: pleural effusion; CSF: cerebrospinal fluid.

The term liquid biopsy is therefore used in contraposition to the traditional surgical tumor biopsy and represents the analysis of cancer biomarkers in tumor-derived material isolated typically from the bloodstream “tumor circulome” [13] or other biofluids such as urine, saliva, cerebrospinal fluid (CSF), pleural effusion (PE), or bile of cancer patients [8]. The analytes used for liquid biopsy include circulating tumor cells (CTCs), cell-free DNA (cfDNA), proteins, metabolites, extracellular vesicles, and cell-free RNA [10], [14]. Regarding the nature of the biomarkers it will depend on the analyte, the type of tumor and the clinical application of the liquid biopsy [8] and include, somatic point mutations, deletions, amplifications, gene-fusions, DNA-methylated marks, tumor-specific miRNAs, proteins or metabolites, etc (Figure 1). Based on the analyte and the biomarker, different technologies are applied to identify and characterize them [12]. Noteworthy, the revolution of Next Generation Sequencing (NGS) technologies has enormously boosted the capabilities of nucleic acid-based liquid biopsies [11].

In 2013, the U.S. Food and Drug Administration (FDA) approved the first liquid biopsy test, the CellSearch^®^ CTC enumeration platform (K073338-FDA) intended to monitor advanced metastasic breast, colon, and prostate cancers based on CTCs counts [15]. Three years later, in 2016, the first cfDNA-based liquid biopsy test was approved. A companion diagnostic test to detect epidermal growth factor receptor (EGFR) mutations in the circulating tumor DNA (ctDNA) of patients with non-small cell lung cancer (NSCLC) who may benefit from targeted therapy with erlotinib and osimertinib [16].

However, many limitations remain unsolved before the routine clinical implementation of liquid biopsies in the field of oncology. Besides the optimization and standardization of protocols to isolate the analytes, there are still open questions such as the complete understanding of tumors behavior and shedding dynamics that challenge the full exploitation of liquid biopsy as a new tool for cancer management. In this still growing scenario, and aiming at accelerating the development, validation, and clinical use of liquid biopsy assays, a collaborative consortium between public, industry, academia, and regulatory agencies was established in both Europe (Cancer-ID; https://www.cancer-id.eu/) and the United States (BloodPAC; https://www.bloodpac.org/).

Here, we review the latest advances of liquid biopsy technology describing the methodologies used to isolate the analytes and to identify the biomarkers, with special focus on CTCs and cfDNA. We also describe the applications of the liquid biopsy in the management of patients with cancer, from the early detection of cancers to treatment guidance in patients with advanced tumors. Finally, we also discuss some current limitations and still relevant questions in the field.

## Types of analytes in cancer patients

Different types of tumor components can be detected in liquid biofluids of cancer patients. As mentioned above, these tumor analytes include CTCs, cfDNA, proteins, metabolites, extracellular vesicles, and cell-free RNA [10]. However, and common to all of them, the tumor analyte is surrounded by a vast majority of non-cancer analytes shedded by normal cells, hindering the isolation and detection of the tumoral fraction of interest. In this scenario, the development of improved sensitive technologies has boosted the interest on this field allowing the use of liquid biopsies as a minimally-invasive source of neoplastic material for molecular analyses. Each analyte has its own advantages and disadvantages, and will provide different information. In some cases, their mere isolation can be already informative or they can be used as the starting material for biomarker detection. Notably, the existence of a proportion of non-shedding tumors (20–58%) has been reported [17], [18]. Therefore, patients with these tumors would not benefit from liquid biopsy advances, and moreover they would increase the false negative rate. However, these affirmations would need to be revisited after the development of more sensitive techniques, as detection sensitivity could be behind this lack of tumor analytes.

Historically, circulating tumor-derived protein-based tests have been the gold-standard approach for screening and guidance of different types of cancers. While widely used, their major and always questioned limitation has been the overdiagnosis [19]. Current investigations are focused on the identification of protein signatures instead of single proteins to overcome the lack of specificity [20] and on improving the detection approaches [21]. Tumor derived-extracellular vesicles are lipid-bilayer-enclosed nanovesicles secreted by tumor cells carrying proteins and nucleic acids [22]. They can be found in almost every biofluid, especially in blood. It has been reported that their levels are increased in different types of cancers [23]. Moreover, by analyzing their content researchers have for instance found that exosomal glypican-1 (GPC1) protein distinguished patients with pancreatic cancer with absolute specificity and sensitivity from healthy subjects [23]. Exosomal *KRA*S and p53 mutations [24] or specific exosomal microRNA signatures [25] were detected in pancreatic cancer patients. The different types of RNA (mRNAs, miRNAs, and lncRNAs) are also released by tumor cells into the bloodstream. However, RNA is a relative unstable molecule and its detection is normally associated with extracellular vesicles. Several examples of exosomal RNA biomarkers in cancer have already been reported [26], [27]. Nevertheless, among tumor liquid biopsy analytes, research on CTCs and cfDNA has experienced an exponential growth in the last decades. Therefore, a detailed revision of these two analytes is presented in the next sections.

## Circulating tumor cells (CTCs)

CTCs are a population of cells that have detached from the tumor mass and circulate in the bloodstream. We have to travel one hundred and 50 years ago for the first documented observation of CTCs. It was in 1869 when the Australian physician Thomas R. Ashworth observed cells that closely resembled the primary tumor in the blood of a man who died due to a metastatic cancer [28]. Nevertheless, it was not until the late 90s of the 20th century when the boom of CTC started, after Racila and colleagues developed the later widely used immunomagnetic enrichment-based methodology for CTC detection. Importantly, they also demonstrated that CTCs exist early in disease and pointed out the potential use of these cells for monitoring treatment responses and recurrences [29], [30].

### CTCs sources and characteristics

The dissemination of tumor cells into the circulatory system is the first step in the metastatic event [31]. Although the mechanisms implicated in this process are still under investigation, it is known that CTC intravasation can occur through a passive release from the primary tumor or through an active intravasation associated to an epithelial-to-mesenchymal transition (EMT) [32]. Once there, if cells survive and spread, they might establish a separate secondary tumor site in a new host organ [31]. However, metastasis is a highly ineffective event, and most of these cells die in circulation owing to trauma, oxidative stress, and attack by the immune system [33]. Indeed, the presence of CTCs in the blood of a cancer patient is an extremely rare event with one CTC per 1 × 10^6^–10^7^ blood cells depending on the disease state [34] and with a half-life in circulation of less than 2.5 h [35]. CTCs are a highly heterogeneous population, as observed at the genetic, transcriptomic, proteomic, or metabolomic level [36] which could reflect their origin from different clones of the primary tumor [37]. CTCs can be distinguished from the mesenchymal blood cells by the expression of epithelial markers such as the epithelial cell adhesion molecule (EpCam) or proteins of the cytokeratin family (CK8, CK18, and CK19), or even by their epithelial morphology [34]. However, as mentioned above and importantly for the isolation protocols, CTCs may have undergone an EMT transition, losing the epithelial markers and characteristic morphology, and expressing instead known EMT regulators, including transforming growth factor (TGF)-*β* pathway components, vimentin, N-cadherin and the FOXC1 transcription factor [32], [38]. Furthermore, a percentage of the CTC population acquires stem-cell-like properties, and consistently express stem-cell markers, including ALDH7A1, CD44, and KLF4 [39].

Recent studies have reported that CTCs also exist in multicellular clusters. They are extremely rare compared to single CTCs, but possess a greater metastatic potential [40]. These clusters can be directly derived from the tumor through passive shedding or collective migration [40], [41], or can be *de novo* formed within the circulation through the aggregation of single CTCs [42]. CTC clusters are comprised of around 2–50 cells with strong cell-cell contacts. In these multicellular aggregates, tumor cells are accompanied by other non-tumor cells including platelets, immune cells, or cancer-associated fibroblasts, suggested to favor CTC clusters survival [32], [43]. Moreover, the proportion of these clusters may be larger than previously anticipated, and increases during cancer metastasis [44]. These CTC clusters have been reported to exhibit enriched expression of keratin 14 [41], plakoglobin [40], and CD44 [42] as well as mesenchymal markers [32].

### Isolation of CTCs

In the last decades several strategies have been developed for the isolation of single CTCs from blood. These technologies are based either on the biological or on the physical differences existing between CTCs and non-tumor blood cells. However, CTCs isolation is still challenging mainly due to their extreme rarity and heterogeneity [36]. Thus, and despite the wide number of approaches already developed and tested, big efforts are still ongoing in this field trying to solve the limitations of current technologies. In addition to the isolation method, pre-analytical variables such as the type of blood collection tube, time between sampling, and processing or storage temperature must also be considered as they will impact the downstream analysis [45]. Here, we will give a general overview of the most relevant CTC isolation methods, but specific review manuscripts focused on CTC technologies are recommended for a comprehensive description [14], [46], [47], [48], [49].

#### Isolation of CTCs based on their biological properties

Methods based on CTCs biological properties rely on specific biomarkers expressed on the cell surface that allow their capture from the blood sample. Positive CTC selection is carried out using tumor-associated cell surface antigens, generally EpCAM and cytokeratins (CK8, CK18, and CK19). However, and as mentioned above, some CTCs loss these epithelial markers and instead express mesenchymal and stem cells markers [32], [38] drawing a blood sample scenario with several CTCs phenotypes. Therefore, a major limitation of the positive-enrichment methods is the bias imposed by the selection marker, which causes a selective capture of specific CTC subpopulations, while losing others. It is worth mentioning as well that EpCAM-based capture has been reported to isolate circulating epithelial cells in patients with benign colon diseases [50], which could therefore result in overdiagnosis. Negative CTC selection relies on the use of antigens expressed in peripheral blood cells but not in CTCs to capture and deplete non-cancer cells from the sample, leaving intact the CTC cells [51]. In this strategy, CD45 antigen is generally used. Although this method results in lower purity compared with the positive enrichment strategy, heterogeneous CTC subpopulations will be isolated together [51], [52]. To apply the immunoaffinity-based principle two different devices are widely used, consisting on selected antibodies bound either to immunomagnetic beads or to microfluidic chips. An additional drawback of these label-capture methodologies is that the binding of CTCs to the surface of the device can complicate CTC recovery and downstream analysis. Currently the CellSearch^®^ system (Menarini Silicon Biosystems) is the only CTC technology approved by the FDA which combines the positive and negative enrichments. It is intended for the enumeration of CTCs in whole blood by an enrichment based on CD45-, EpCAM+, and cytokeratins 8+, 18+, and/or 19+ [15].

#### Isolation of CTCs based on their physical properties

Methods based on CTCs physical properties rely on the physical differences such as size, density, deformability or electric charge, between CTCs and other blood cells (mainly leukocytes) [47]. CTCs are supposed to be larger in size than normal blood cells [53]. Density of CTCs is similar to nucleate blood cells, lying between plasma and red blood cells after centrifugation. Several reports have determined that CTCs exhibit greater deformability capacity than normal cells [54], [55]. Additionally, electrical features of CTCs have been also applied to discriminate them from non-tumorigenic blood cells using dielectrophoresis [56]. Finally, there are other CTC isolation methods based on their functional features, including their ability to digest cell adhesion matrix, to secrete different proteins or to overexpress the enzyme telomerase [13].

### Usefulness, current limitations and important considerations of CTCs

Despite the numerous platforms developed for CTC analysis and the exponential number of publications in this field, the translation into the clinical practice is still limited. To date, several reports have determined that the mere isolation of CTCs and their count number per mL of blood is already a biomarker itself with potential clinical implications. However, only the CellSearch^®^ CTC enumeration platform is nowadays approved by the FDA as a prognostic predictor, providing reliable information on progression-free survival and overall survival, in metastatic breast [44], colon [53], and prostate [56] cancers. Patients with metastatic disease holding < 5 CTCs per 7.5 mL of blood are more likely to have a better clinical response than those with > 5 CTCs per 7.5 mL of blood [57]. Additionally, the clinical validity of CTC counts to select the first-line treatment for metastatic, hormone-receptors positive, breast cancer patients is currently being evaluated (MATABREAST trial; NCT01710605). However, as discussed by Aceto [58], fluctuations in CTC numbers in patients with comparable disease status, overall burden, and metastatic profile have been observed, calling the clinical utility of CTCs presence and abundance into question. Further knowledge regarding the factors influencing CTC shedding, dynamics, and clearance are still needed to have a complete picture and therefore develop more accurate tools for their interpretation.

Nevertheless, CTCs can be as well the analyte for subsequent biomarker analyses. In this regard, CTCs can provide real-time proteomic, (epi) genetic, genomic, or transcriptomic information of the tumor. For example, a test has been developed to detect nuclear androgen-receptor splicing variant 7 (AR-V7) protein expressions in isolated CTCs to guide treatment selection in patients with metastatic castration-resistant prostate cancer [59]. Methylation profiles of tumor suppressor genes in CTCs correlates with metastatic potential and poorer prognosis [60]. Moreover, a microfluidic western blot system to assess the level of eight proteins in individual CTCs derived from estrogen receptor-positive breast cancer patients has been recently developed [61].

## Circulating cell-free DNA

Small fragments of DNA circulate freely in the peripheral blood of healthy and diseased individuals. In the case of cancer patients, a fraction of these circulating cell-free DNA (cfDNA) molecules correspond to circulating tumor DNA (ctDNA). If we look back, the presence of soluble DNA in the blood was firstly reported in 1948 by Mandel and Metais [62]. However, it was not until 1977 when serum cfDNA levels were associated to cancer patients and the effect of therapy [63]. We had to wait until 1994 for the discovery of the presence of a *KRA*S mutation in the cfDNA extracted from plasma of patients with pancreatic cancer [64].

### cfDNA sources and characteristics

DNA fragments are released into the bloodstream mainly via cell apoptosis, but also through other type of cell death processes such as cell necrosis, pyroptosis, or autophagy, as well as through active cell secretion [65]. cfDNA can originate from both nuclear and mitochondrial sources [66]. These circulating cfDNA fragments are about 166 base pairs in length, which correspond to the length occupied by a nucleosome. Once there, cfDNA clearance occurs through enzymatic degradation (DNase I, plasma factor VII-activating protease, and factor H), renal excretion, and liver and spleen metabolism [67]. The balance between release and clearance determine cfDNA half-life and although it varies between individuals and conditions, it normally ranges from 16 min to 2.5 h [67], [68]. Epigenetic studies have demonstrated that the circulating nucleosome footprints and the cfDNA methylation patterns of healthy individuals strongly correlate with that of lymphoid and myeloid cells [69], [70], pointing out to the hematopoietic system as the predominant source of cfDNA.

In cancer patients, a proportion of these cfDNA molecules also derive from the primary and secondary tumors [64]. Although it was originally thought that the higher level of cfDNA in the blood of cancer patients might be a cancer biomarker itself, it has been since shown that many other conditions result in similar cfDNA increase. In this regard, important points must be considered: i) concentrations of cfDNA vary enormously between individuals and their physiopathological conditions, being increased not only in advanced cancer patients but also in other scenarios including autoimmune diseases, trauma, exhaustive exercise, or pregnancy; ii) in most early stage cancers, the amount of cfDNA is very low, similar to healthy subjects [71]; iii) the fraction of ctDNA fragments in the total cfDNA is very small, varying from less than 0.01% to over 10% according to tumor burden [72] and tumor metabolism [73]. ctDNA fragments are usually smaller than the cfDNA released by healthy cells. A recent study demonstrated that these different fragmentation profiles could be used not only for cancer screening but also for the determination of the tissue of origin of cancers [74].

### Isolation of circulating cfDNA

Although cfDNA can be obtained from different biofluids, to date the majority of studies have been performed with blood as the starting material. This blood is usually collected from peripheral veins; however, it remains unknown whether higher cfDNA yield and ctDNA proportion would change depending on the sampling site. In spite of the exponential focus and advances in this field, we still lack standardized protocols, both for preanalytical sample preparation after blood collection and for plasma cfDNA purification [75]. It should be noted that the variables inherent in these steps may affect the quality of the analyte, and could compromise the interpretation of the final outcome and the comparison between studies.

Several studies have been published aiming at comparing the different methodological options at each single step and recent reviews have put together the obtained information [45], [75], [76] (Table 1). Although both serum and plasma could be used as cfDNA source, plasma is preferred to avoid contamination with genomic DNA derived from white blood cells lysis [77]. Thus, blood should be collected in EDTA anticoagulant tubes and processed within the first 4–6 h after sampling. If quick processing is not possible, specialized cell-stabilizing tubes are available that prevent leukocytes lysis [78]. Evidence suggests that a two-centrifugation steps protocol -first one at low, and the second at high speed- is optimal for plasma recovery and subsequent cfDNA isolation [75]. An optimal cfDNA extraction method should purify all cfDNA fragments avoiding genomic DNA, and minimize the presence of PCR inhibitors. Several methods are commercially available for cfDNA-specific extraction based on either magnetic beads or silica column-based membranes. Some of these kits are manual, whereas others use automated systems that minimize sample handling. Although the variations between the different kits and protocols are unclear, the choice of the extraction method can result in different cfDNA yield [76]. The concentration of cfDNA can be determined using different methodologies ranging from fluorometer approaches to PCR-based assays [75], [76].

**Table 1:** Methodological variables in processing and analysis of cfDNA.

Protocol steps	Variables/Considerations
1. Blood collection [75]	Specimen type: plasma is preferred to serum to avoid contamination with genomic DNA.Collection tube: it will depend on the time to processing: EDTA-tubes when plasma is processed within the first 4–6 h after sampling. Cell-stabilizing tubes when plasma quick processing is not possible (stable up to ∼7 days).
2. Centrifugation for plasma isolation [75]	Protocol: a two-centrifugation steps protocol is required. First at low (∼2500 *g*), second at high speed (∼14000 *g*). Apparently, temperature (room temperature or 4 °C) is not critical. *After the first centrifugation, plasma can be frozen at −80 °C for later processing.
3. cfDNA extraction [75], [76]	Method: different cfDNA isolation kits (from Qiagen, Promega, Applied Biosystems, Zymo Research, Norgen Biotek, EpiGenTek, among others) are available. Some considerations to take into account: Analyte: extraction of cfDNA or total nucleic acids (cfNA) Handling: manual or automatized Technology employed: silica-based membrane, magnetic particles or beads, spin column processing, among others. *If plasma was frozen at −80 °C, thawing must be done slowly in ice.
4. cfDNA quantification [75], [76]	Method: quantification of cfDNA yield can be performed by: Fluorometry (Qubit, Quantus, Quant-iT PicoGreen assays) PCR: qPCR, Digital PCR
5. Biomarker analysis [12], [81]	Biomarker features: mutation, amplification, fusion, methylation, among others. Approach: according to the biomarker's nature , additional processing steps may be required (for instance, DNA bisulfite treatment for methylation detection). Specific candidate analysis (qPCR, Digital PCR, among others). High throughput analysis: Next Generation Sequencing (NGS) (targeted, whole exome sequencing, whole genome sequencing, among others).

### Biomarkers in cfDNA, current limitations and important considerations

As the circulating cfDNA released by tumor cells preserves the characteristics of the cell of origin, the identification of specific genetic and epigenetic tumor alterations in this liquid biopsy analyte has emerged as a promising tool with immense potential in cancer management. As mentioned before, the pool of ctDNA would better reflect the entire tumor epi/genetic picture rather than the traditional tumor biopsy, recapitulating tumor heterogeneity. However, analysis of cfDNA for cancer management still has several drawbacks. We can highlight the low amounts of mutant fragments in the total cfDNA sample which limits the detection success giving rise to false negative results [72]. In addition, it has been recently demonstrated that mutations can derive from clonal hematopoiesis rather than from tumor cells, thus giving rise to false positive results [79]. Indeed, according to a recent publication, a high percentage of cfDNA mutations found in both controls and cancer patients originate from clonal hematopoiesis, highlighting the importance of processing in parallel matched cfDNA and white blood cell DNA [80]. Caution is therefore needed when selecting biomarkers and interpreting cfDNA outcomes. The methodologies used to study the tumor material can be divided into targeted or untargeted strategies. When specific biomarkers are interrogated, digital PCR-based technologies such as droplet digital PCR (ddPCR) and BEAMing (beads, emulsion, amplification, and magnetics) show very high sensitivity, being fast and relatively inexpensive [81]. When a variable number of specific candidate biomarkers are interrogated, NGS-based panels have been designed, such as Tam-Seq (Tagged AMplicon deep sequencing), Safe-Seq (safe sequencing system) or CAPP-Seq (Cancer Personalized Profiling by deep sequencing) (review in ref. [12], [81]). To identify novel biomarkers, NGS technologies need to be applied. They have the limitation of lower sensitivity and higher cfDNA input [81]. Besides the enormous amount of published studies in the field and the huge efforts done by researchers in the last decades, to date only three cfDNA-based tests have been approved by the FDA (Table 2). As mentioned before, the first approved cfDNA-based liquid biopsy was a companion diagnostic test. The Cobas EGFR Mutation test v2 (Roche Molecular System, Inc), relies on real-time PCR detection of 42 mutations in exons 18, 19, 20 and 21 in the *EGF*R gene including L858R, exon 19 deletions, and T790 M mutation using plasma cfDNA within 4 h. The approved application of this test was to identify among patients with advanced or metastatic non-small cell lung cancer (NSCLC) those with an EGFR mutation, being therefore suitable for EGFR-targeted therapy [16]. Other FDA approved cfDNA-based assay is the Epi proColon test (Epigenomics AG) as a screening test for colorectal cancer (CRC). This test is designed for the detection of methylated *SEPT9* in bisulfite converted plasma cfDNA by real-time PCR. The whole process takes around 10 h. Methylation of *SEPT9* promoter has been associated with CRCoccurrence [82]. For their straightforward application, the corresponding companies (Roche Molecular System and Epigenomics AG) have developed kits including all the material and instructions needed from sample preparation to interpretations of the results. Whereas a positive result is very informative and useful for patients' management, a negative result in these liquid biopsy tests should be considered inconclusive. Finally, the multiplex PCR and NGS based ClonoSEQ kit, which gained FDA approval in September 2018, is intended to follow disease burden changes over time in response to treatment or during remission in both acute lymphoblastic leukemia or multiple myeloma patients [83]. It assesses and quantifies a panel of immunoglobulin receptor gene sequences and frequently translocated regions of the genome being able to detect a single cancer cell among a million cells [83].

**Table 2:** Liquid biopsy FDA-approved tests.

Kit/test	Company	FDA Status	Alteration detected /technology	Application	Other information
T Cell Search Circulating Tumor Cell Kit [15]	Menarini Silicon Biosystems	FDA-approved (August 2013)	Enumeration of circulating tumor cells (CTC) of epithelial origin: EpCAM positive enrichment and detection of cytokeratins 8, 18, and/or 19.	Prognosis of metastatic breast, colorectal, or prostate cancer	The sensitivity of this kit will depend on the positivity of CTCs for EpCAM surface marker as well as cytokeratins 8, 18, and/or 19.
Epi proColon DNA-methylation blood test [82]	Epigenomics AG	FDA-approved (April 2016)	Detection of methylated cytosine residues in the *SEPTIN9* gene in ctDNA by real-time PCR	Screening for colon cancer	The company has developed a kit to assess *SEPTIN9* methylation that will be completed in around 32 h. According to the literature, its sensitivity ranges from 69-72%.
Cobas EGFR Mutation Test v2 [16]	Roche Diagnostics	FDA-approved (June 2016)	Detection of 42 defined mutations in the epidermal growth factor receptor (*EGFR*) gene by real-time PCR	Guiding treatment selection in non-small-cell lung carcinoma	This test allows for detection of mutations in cfDNA in less than 4 h (12). 75% of sensitivity and 98% of specificity.
ClonoSEQ [83]	Adaptiv Biotechnologies	FDA-approved (Septembre 2018)	Detection of immunoglobulin receptor gene sequences and frequently translocated regions by multiplex PCR and next-generation sequencing (NGS).	Detection of minimal residual disease in acute lymphoblastic leukemia or multiple myeloma	ClonoSeq detects a single cancer cell among a million cells. Data processing will take between 7 and 14 days.

## Applications and current limitations of liquid biopsies for cancer

**Figure 2:**
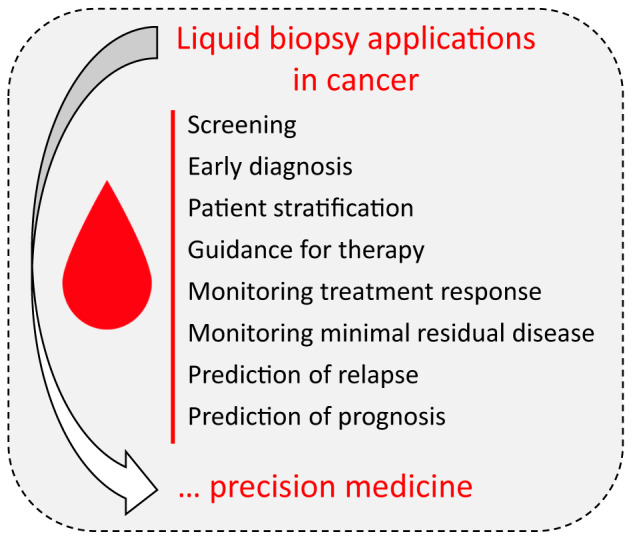
Summary of liquid biopsy applications for cancer management.

Despite the great advances in cancer management, this disease is still one of the world's most pressing health care concerns. Current important challenges include early diagnosis, accurate patient stratification and treatment selection, monitoring response to therapy and detection of minimal residual disease, and risk of relapse (Figure 2). To address all these concerns, liquid biopsy-based tools are continuously showing increased potential and, besides of researchers, have attracted the attention of investors [8], [10]. However, hard work is still needed as to date few tests have received the FDA approval or FDA Breakthrough Device Designation (Table 2 and 3).

**Table 3:** Selected liquid biopsy tests within the FDA's Breakthrough Devices Program.

Kit/test	Company	Technology/application	FDA Status
Foundation One Liquid test	Roche Foundation Medicine	NGS test to detect clinically relevant indels, substitutions, CNV and selected genetic rearrangements in 70 oncogenes for companion diagnostic	FDA granted Breakthrough Device Designation (April 2018)
Multicancer early detection test	Grail	NGS blood test analyzing ctDNA methylation patterns for detecting multiple cancer types	FDA granted Breakthrough Device Designation (May 2019)
Guardant 360	Guardant Health	ctDNA test of mutations (73 genes), amplifications (18 genes), fusions (6 genes), indels (23 genes), to guide treatment selection in non-small-cell lung carcinoma	FDA granted Breakthrough Device Designation (May 2019)
Resolution HRD	Resolution Bioscience	NGS assay to detect sequence variations in genes associated with homologous recombination deficiency (HRD). Companion diagnostic for prostate cancer	FDA granted Breakthrough Device Designation (May 2019)
Cancer SEEK	Thrive Earlier Detection	Multianalyte test that combines multiplexed PCR detection of mutations in ctDNA at 1,933 loci with measurements of validated protein biomarkers to diagnose eight common cancer types	FDA granted Breakthrough Device Designation (May 2019)
ExoDx Prostate IntelliScore (EPI) test	Bio-Techne	Urine exosome-based genomic test for the diagnosis of prostate cancer	FDA granted Breakthrough Device Designation (June 2019)
Ivy Gene CORE Test; IvyGene DX Liver Test	Laboratory for Advanced Medicine (LAM)	Hyper-methylated ctDNA from multiple gene targets. To confirm the presence of breast, colon, liver, and lung cancers as early as stage 1	FDA granted Breakthrough Device Designation (September 2019)
[88], [89], [90]			

Evidence show that the efficacy of any anti-tumoral therapy including conventional chemotherapy, targeted therapy, or immune checkpoint inhibitors significantly improves when the tumor burden is low, making urgent the implementation of early detection methods. In this regard, liquid biopsy appears as a promising technique for cancer screening and early diagnosis. However, some limitations need to be solved before its incorporation in the clinical practice [11]. As mentioned above, one of the major obstacles in liquid biopsy-based early diagnosis is that biofluids of these patients harbour almost undetectable tumor analytes. In addition, the identification of the organ of origin, the high rate of false positive (overdiagnosis) and false negative (underdiagnosis) results due to both technical (biomarkers selection, sample processing, limited sensitivity), and biological (non-shedding tumors or clonal hematopoiesis of indeterminate potential) factors must be upgraded [11], [17], [79]. In this sense, the use of multiple types of biomarkers combining different analytes appears as a promising alternative to increase sensitivity and specificity [9], [84]. For instance, the CancerSEEK blood test is able to detect eight common types of cancer by assessing eight protein biomarkers and tumor-specific mutations in circulating cfDNA found in blood samples [84].

Once cancer diagnosis has been confirmed patient stratification and treatment decisions will depend on an accurate molecular profiling of the tumor. In this regard, besides being minimally-invasive, liquid biopsies have the advantage over tissue biopsy of recapitulating a more complete tumoral picture, theoretically allowing a more precise analysis. In this scenario, efforts are being made to identify actionable mutations or gene expression patterns in liquid biopsy analytes that could guide therapy. For example, as mentioned before, the FDA approved Cobas EGFR Mutation test v2 (Roche Molecular System, Inc) aims at distinguishing NSCLC patients that will benefit from EGFR-targeted therapy [16] and the Guardan360 (Guardant Health) examines a 73-gene panel in cfDNA to help NSCLC treatment selection. Similarly, ongoing research suggests that exosomal PD-L1 levels may constitute a predictor for anti-PD-1 therapy [85].

Monitoring treatment response and early detection of recurrence is essential to gain time for a quick second shot against cancer. Liquid biopsies as well hold promise in this context, with the great advantage of easily allowing longitudinal sample collection for a precise and continued follow-up. Tumor-derived analytes should decrease after complete surgical resection or during the evolution of a curative treatment. If specific tumor features or CTCs have been identified in the diagnostic phase, they could be followed-up as a warning alarm. In agreement, it has been reported that persistent detection of tumor analytes predicts high risk of relapse in different types of tumors [72], [86], [87]. Remarkably, the FDA-approved ClonoSEQ test is intended to detect minimal residual disease in acute lymphoblastic leukemia or multiple myeloma patients after completion of initial therapy [83].

## Concluding remarks and open questions

In the last decades, liquid biopsy has emerged as a promising minimally-invasive tool for cancer management. Behind the term “liquid biopsy” a wide bulk of concepts are included since it encompasses different biofluids, analytes, biomarkers, technologies, and applications. Researchers are progressing at giant steps and are generating new knowledge that is forming the basis for the development of liquid biopsy tests. However, the lack of homogeneity in the protocols and the competitiveness in the field, makes it difficult to conclude with firmness whether we are about to implement those liquid biopsy assays in a real-world clinical setting. Moreover, relevant and unsolved questions still remain open. After a quick look at the literature, several protocols with a number of different variables are found when one tries to elucidate how to isolate a liquid biopsy analyte. Would it be possible to develop a unique standard isolation protocol for each component of the tumor circulome? This would help regarding the standardization and interpretation of results. The majority of the diagnostic studies have been performed comparing cancer patients versus healthy controls, but in a true clinicalpractice setting screenings would be done in patients with different non-tumoral pathological conditions. Will tests maintain their calculated specificity? Moreover, and as mentioned, the existence of non-shedding tumors has been described [17], [18]. Do these tumors really exist and in which percentage? If yes, they may increase the number of underdiagnosed cases and complicate the interpretation of tests. Where should we establish the sensitivity threshold of a liquid biopsy test to be considered useful? Regarding overdiagnosis, and before considering a test valid, which should be the size and the characteristics of the individuals tested as controls? In spite of these opened questions, the studies reviewed here underline the potential of liquid biopsy for cancer precision medicine. With researchers all over the world focused on accelerate liquid biopsy knowledge and applications, soon these questions will be solved and the translation to the clinical practice of these minimally-invasive technologies will revolutionize cancer patient management.
